# Seropositivity for *Trypanosoma cruzi* in domestic dogs from Sonora, Mexico

**DOI:** 10.1186/s40249-017-0333-z

**Published:** 2017-09-05

**Authors:** Minerva Arce-Fonseca, Silvia C. Carrillo-Sánchez, Ramón M. Molina-Barrios, Mariana Martínez-Cruz, Jesús R. Cedillo-Cobián, Yuly A. Henao-Díaz, Olivia Rodríguez-Morales

**Affiliations:** 1 Department of Molecular Biology, National Institute of Cardiology “Ignacio Chávez”, Juan Badiano No. 1, Col. Sección XVI, Delegación Tlalpan, 14080 Mexico City, Mexico; 2 Department of Agronomics and Veterinary Sciences, Technological Institute of Sonora, 5 de Febrero 818 Sur, Centro, 85000, Cd Obregón, Mexico City, Sonora Mexico

**Keywords:** Chagas disease, Epidemiology, Reservoir, US-Mexico border dogs, *Trypanosoma cruzi*, Mexico

## Abstract

**Background:**

Chagas disease is an important health problem in Latin America due to its incapacitating effects and associated mortality. Studies on seropositivity for *Trypanosoma cruzi* in Mexican dogs have demonstrated a direct correlation between seropositivity in humans and dogs, which can act as sentinels for the disease in this region. The objective of this study was to determine the seropositivity for *T.cruzi* infection in dogs from Sonora, a northern borderstate of Mexico.

**Methods:**

Responsible pet owners were selected at random from an urban area of Empalme municipality, Sonora, Mexico, and from there, 180 dog samples were collected. Anti-*T. cruzi* antibodies were determined using the enzyme-linked immunosorbent assay (ELISA) method. Reactive ELISA sera were processed by indirect immunofluorescence to confirm the presence of anti-*T. cruzi* antibodies. For the statistical analysis, chi-square tests were conducted.

**Results:**

Dogs’ sera showed a seropositivity rate of 4.44%. The rate of seropositivity was not associated with the dogs’ age, sex, or socioeconomics pertaining to the geographical area. One sample (1/180, 0.55%) showed the acute state of the disease.

**Conclusions:**

The study found a presence of anti-*T. cruzi* antibodies in dogs in this area, which suggests vector transmission. There is a need for active surveillance programs throughout the state of Sonora and vector control strategies should also be implemented in endemic regions.

## Background

Chagas disease (CD) is caused by the protozoan *Trypanosoma cruzi*. Because the parasite is distributed in areas ranging from the southern United States [1] to Argentina and Chile, the disease is commonly known as American trypanosomiasis*.* Epidemiological data from 2010 estimated that there were about 5,742,167 people infected in Latin America and 70,199,360 at risk of acquiring infection. Argentina, Brazil, and Mexico were the three countries with the highest estimated number of infected people (1,505,235; 1,156,821; and 876,458, respectively) [2]. This disease has been one of the biggest public health problems in Latin America due to its incapacitating effects and associated mortality [3]. Increased international migration has resulted in the disease spreading to non-endemic areas, including Canada, Europe, Asia, and Oceania. Although vector-borne transmission cannot occur outside of America (as the vector is not present), the disease can spread via vertical transmission, blood transfusions, and organ transplantations in non-endemic countries [4–6].

In Mexico, there are no official programs for vector control. In addition, there is no consensus on diagnostic methods for acute and chronic phases of CD, and trypanocidal therapy is rarely administered to chronic patients because the availability of trypanocidal drugs is restricted, and physicians only treat the cardiac or digestive symptoms, as they consider drugs are ineffective in this stage of the disease and have undesirable side effects. The current prevalence of the disease is unknown because there are no official cases reported. However, based on the percentage of seropositivity published by Cruz-Reyes and Pickering-López in 2006 [7], and considering there are 112.3 million inhabitants in Mexico (as according to the 2010 National Population Census [8]), it is possible to estimate that 5.5 million people are at risk of acquiring the *T. cruzi* infection in the country [9].

The transmission cycle includes mammalian reservoirs of the parasite and triatomine species as vectors, which colonize domestic and peridomestic environments. Dogs are considered the most important domestic reservoir of the parasite in the dynamics of *T. cruzi* infection because dogs are an important food source for triatomine bugs and they can also eat infected insects. Thus, it has been demonstrated that infected dogs increase the risk of transmission inside human dwellings [10]. From the veterinary point of view, it is also important to consider that dogs are susceptible to acquire American trypanosomiasis characterized by heart conditions, such as electrical conduction disturbances, and ventricular and supraventricular arrhythmias, as well as secondary signs such as ascites, respiratory distress, thoracic effusion, and cyanosis [11, 12].

It is believed that over 96% of CD transmission occurs by the vector route [13]. Mexico has one of the most diverse populations of triatomine bugs, with 32 documented *T.cruzi*-infected species, establishing them as potential vectors of CD [14]. Few national studies have estimated the presence of *T. cruzi* in domestic reservoirs in Mexico. In the state of Sonora, there are no reports about seropositivity for *T. cruzi* infection in dogs, despite having sufficient case reports in other countries indicating that the presence of this mammal in households can increase the human risk of contracting the disease by up to five times [15].

Studies describing the seropositivity rate in dogs in Mexico (see Table 1) have demonstrated a direct correlation with the presence of antibodies against *T. cruzi* in humans [10, 16-26]. The high seropositivity rate of *T. cruzi* infection in dogs from different regions in the country suggests that dogs are a potential domestic reservoir and are thus potential perpetuators of the *T. cruzi* transmission to humans. In Sonora, there is no information about prevention and control programs relating to CD, despite it being an endemic area. This is largely because a lack of information and under-diagnosis overlaps the actual incidence of the disease, which promotes the use of diagnostic tools in reservoirs in order to project the impact of the prevalence of infection on human population.

**Table 1:** Seroepidemiology Against *Trypanosoma cruzi* in Mexican Dogs, 2001–2014

State (City or Municipality)	Seroprevalence in dogs (%)	Seroprevalence in humans (%)	Methodology	Reference
Morelos (Cuernavaca) Puebla (Puebla)	8.8 24.2	ND ND	ELISA	[16]
Estado de México (Tlalnepantla)	1.2	ND	ELISA WB	[17]
Puebla (Palmar del Bravo)	10.6	4	ELISA IIF	[18]
Estado de México (Tejupilco and Toluca)	21 17.5	7.1 ND	IHA IIF ELISA	[10]
Yucatán (Tunkas and Mérida)	9.8 17.3	ND ND	IIF WB PCR	[19]
Chiapas (Tuxtla Gutiérrez)	4.5	ND	IIF WB	[20]
Yucatán (Mérida)	34	8	ELISA IIF WB	[21]
Campeche (Campeche)	7.6	0.1	ELISA IIF WB	[22]
Estado de México (Tejupilco)	24.5	ND	IHA ELISA	[23]
Morelos (Puente Pantitlán)	24.2	1.2	ELISA IIF	[24]
Estado de México (Toluca)	0.34	ND	IHA ELISA	[25]
Jalisco (Teocuitatlán)	8.1	ND	ELISA WB	[26]
Sonora (Empalme)	4.44	ND	ELISA IIF	This study

*ND* Not determined, *ELISA* Enzyme-linked immunosorbent assay, *WB* Western blot test, *IIF* Indirect immunofluorescence test, *IHA* Indirect hemagglutination test

This study sought to determine the presence of antibodies against *T.cruzi* in domestic dogs (*Canis lupus familiaris*) in Sonora, Mexico. This is relevant as a public health issue because this study is the first report about seropositivity in a major domestic reservoir of the disease in a region which, despite being considered endemic, has not seen any governmental actions for controlling and preventing the disease. Sonora is now added to the list of states in Mexico (8 out of 32) that have had studies conducted on the seropositivity in dogs as the main domestic reservoir of CD.

## Methods

### Study area and period of sampling

**Fig. 1:**
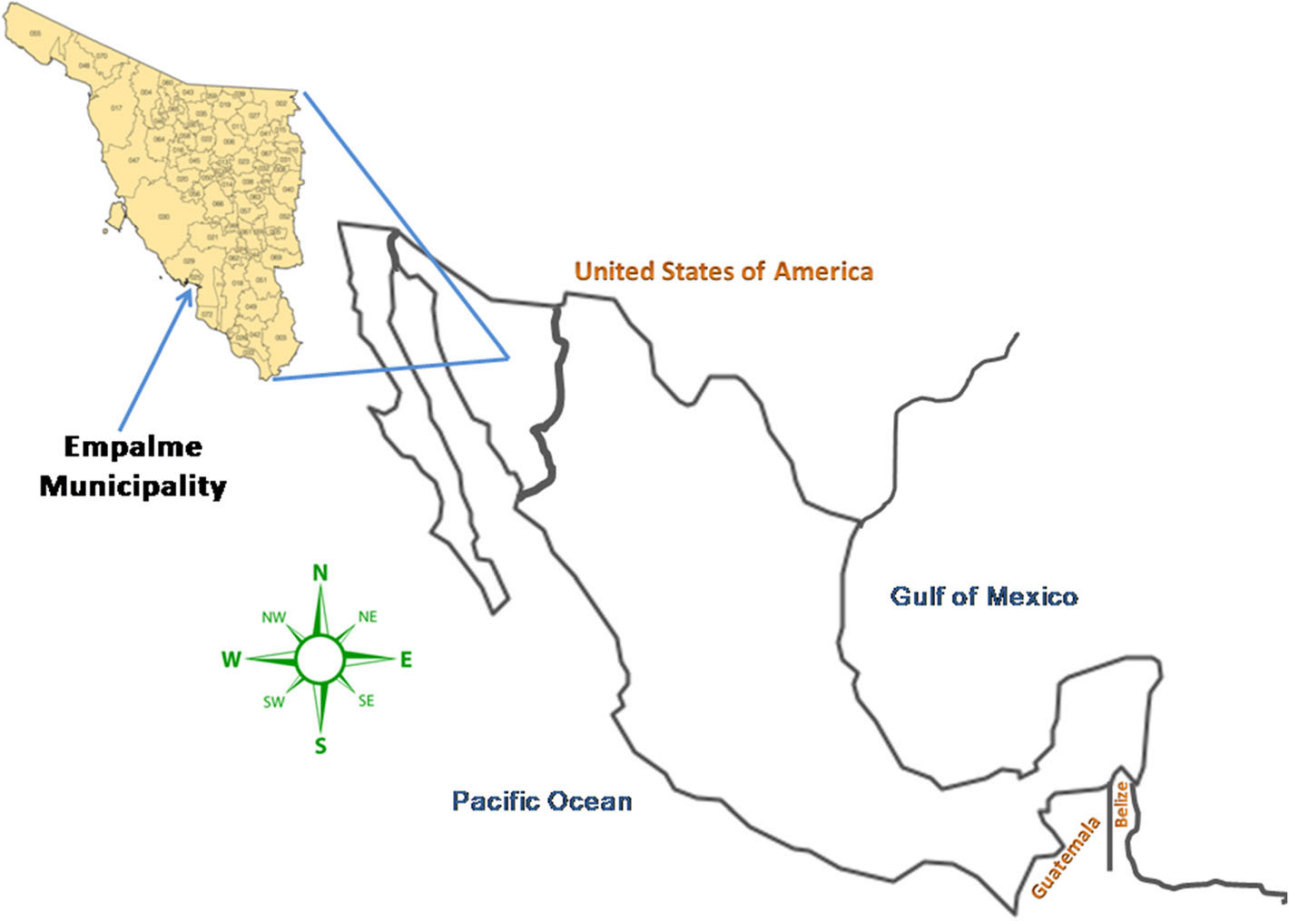
Geographical location of the study area. Map of Mexico showing the Empalme municipality in the state of Sonora

The study was conducted in the Empalme municipality (110°48′51″ west latitude and 27°57′42″ north longitude), in the state of Sonora, Mexico (see Fig. 1). Empalme has an area of 503 km^2^, a height above sea level of 1 m, an average temperature ranging between 17 °C and 29 °C, and a population of 54,131 inhabitants in 2010, with an average of 3.8 people per dwelling.

There are three well defined geographical zones that correspond with the population’s socioeconomic status: 1) a poor area in the northwest near the municipal garbage dump, which is characterized by poor housing and minimum municipal services; 2) the central region, which is characterized by people of median income; and 3) an affluent area in the east and southern regions, which has a population with a high economic status and good housing conditions and municipal services [27]. Sampling was performed during the spring and summer months (May to August) in 2013.

### Study population

Samples were collected from an urban area of the Empalme municipality by selecting dogs at random, regardless of their overall health, age, breed, or sex. The only inclusion criteria were that they resided in the municipality and were pets of responsible owners, defining them as people who fed their dogs and gave consent for their dogs to be sampled.

The sample size was dictated by a specific statistics formula for the detection of a disease in a population with a confidence level of 95%. If in our sample size no animal comes out positive it would mean that the population is free of the disease. Taking into account a 95% confidence level, an estimated 1.5% of dogs with CD, and 800 responsible dog owners as the expected total population, the number of required samples was determined to be at least 169. In this study, a total of 180 samples are reported on.

### Sample collection

Blood samples were taken directly from the cephalic vein. To raise this vein, the dorsal region of the foreleg was constrained and disposable 5 ml syringes with 22G needles (Protec ®, Estado de México, Mexico) on small animals were used to puncture the vein. A double-ended needle plastic plunger and Vacutainer blood collection tubes (BD Vacunainer ® Becton, Dickinson and Company, NJ, USA) without anticoagulant were used on larger ones. Subsequently, the clot was removed and the serum was separated by centrifugation at 3000 rpm for seven minutes. Sera were frozen at −20 °C until use.

### Control sera

Anti-*T. cruzi* positive sera from the acute phase of experimental CD were obtained from two Náhuatl dogs. The Náhuatl breed is a Mexican canine inbred, verified, certified, and validated as a biological model by the Mexican Association of Animal Science Laboratory, dated November 25, 2015. The dogs were infected intraperitoneally using a 1.5 × 10^6^ metacyclic trypomastigotes per animal of the Ninoa strain (MHOM/MX/1994/Ninoa), obtained from the urine and feces of infected triatomines and resuspended in saline solution. Animal handling was done following the International Guiding Principles for Biomedical Research Involving Animals and the *Norma Oficial Mexicana* (NOM-0062-ZOO 1999): Technical Specifications for the Care and Use of Laboratory Animals.

In previous studies, we obtained anti-*T. cruzi* positive control sera of the chronic phase of CD from dogs inoculated with 2 × 10^5^ metacyclic trypomastigotes of the Ninoa strain (MHOM/MX/1994/Ninoa) [28].

### Determination of *T. cruzi* anti-immunoglobulin G and M (IgG and IgM) by ELISA

Anti-*T. cruzi* antibodies in the 180 dogs were determined using the enzyme-linked immunosorbent assay (ELISA) method described previously [28], with a total extraction of *T. cruzi* INC-9 isolates (MHOM/MX/2001/INC9) as an antigen and Goat anti-Canine IgG or IgM Secondary Antibody (Novus Biologicals®, CO, USA), as according to the manufacturer’s instructions. The mean optical density of seronegative healthy domestic dogs plus three standard deviations were fixed to set the cut-off. All healthy seronegative dogs had values classified as negative after the cut-off value was set.

### Indirect immunofluorescence assay

Reactive ELISA sera were processed by indirect immunofluorescence (IIF) using a method described previously [28]. Positive fluorescence was defined as the detection of green fluorescence on parasites andwas labeled with + /+ + + +, according to the fluorescence degree observed using a positive control as the reference point.

### Statistical analysis

The associations between the dogs’ ages and sex, and the socioeconomic status pertaining to the geographical area where the dogs live, and the specific antibodies to CD were calculated using chi-square tests with the SPSS ® program version 15.0 (IL, USA). The level of significance was set at *P* ≤ 0.05.

## Results

More than 95% of the dogs sampled for this study live in homes non-delimited by fences made of solid material; the dwellings only have wooden fences or are surrounded by fences with razor-sharp concertina wire allowing the dogs to roam outside freely. The animals have access to the inside of the house without any restriction; however, they spend the night outdoors in improvised places such as barns, shed or roofed-over areas that protect them from inclement weather. The dogs roam freely on the streets near their homes at any time of day. They do not receive veterinary care and are not covered by a program of preventive immunization. The presence of domestic birds (hens) and/or cats was observed in all dwellings.

The reactive (positive) samples tested by IgG detection using the ELISA method were 19/180 (10.56%). Of these 19 ELISA reactive samples, 8 (4.44%) were confirmed as positive when IIF was used as a secondary test. Thus, the seropositivity for *T. cruzi* in dogs in Empalme was determined to be 4.44% (8/180) by the two tests used (see Table 1).

In order to assess the risk of people being exposed to infected bugs in peridomestic areas based on the detection of IgM in the canine population acting as reservoirs and sentinels, only one sample showed IgM levels above those found in positive control dogs infected experimentally. These data suggest that 0.55% of dogs sampled showed the acute stage of the disease (Data not shown).

Of the positive samples diagnosed using both tests, five (62.5%) were from male dogs and three (37.5%) were from female dogs; seven (87.5%) were mongrels and one (12.5%) was a bred dog. The eight positive samples confirmed by IIF were sera from puppies (between 0 and 18 months old) and adult dogs (between 18 and 48 months old). Younger dogs compared to older dogs as well as male dogs (5/8) were more likely to be positive for CD. However, the presence of specific antibodies for Chagas infection in dogs from this urban area was not associated with the dogs’ age or sex because no statistical differences among these risk factors and the seropositivity rate were found (see Table 2). The geographic area according to the socioeconomic status also did not represent a risk factor for seropositivity for *T. cruzi* infection (8/180) in dogs from this area (see Table 2) (*P* ≤ 0.05).

**Table 2:** Factors associated with the prevalence of Chagas Disease in Empalme, Sonora, Mexico, 2015

Factor	Number of dogs *n*/180(%)	Chagas disease in dogs by ELISA and confirmatory IIF tests *n*/180 (prevalence %)
Age		
Puppy	106 (58.88%)	4 (2.22%)
Adult	68 (37.77%)	4 (2.22%)
Geriatric	6 (3.33%)	0 (0%)
Gender		
Female	71 (39.44%)	3 (1.67%)
Male	109 (60.56%)	5 (2.78%)
Economic status by geographic area		
Low	56 (31.11%)	5 (2.78%)
Medium	78 (43.33%)	3 (1.67%)
High	46 (25.55%)	0 (0%)
Total dogs	180	8
Percentage	100%	4.44%

*ELISA* enzyme-linked immuno assay, *IIF* indirect immunofluorescence test

*Difference was considered as significant when *P ≤* 0.05 by Chi-Square test

## Discussion

Large numbers of wild and domestic animals act as reservoirs for *T.cruzi* infection, however, dogs play a very important role in the prevalence of the disease in the human population because they are the most common mammal found in homes and because they usually come into close contact with their owners. Although cats, pigs, cattle, and other mammals that serve as reservoirs are also in constant contact with humans, it has been demonstrated that the presence of dogs in homes can increase the human risk of acquiring parasitosis by up to five times. It is therefore crucial to study the course of *T. cruzi* infection in this species, which can act as sentinels for CD in this geographic area [15, 28].

Numerous epidemiological studies have been conducted in dogs from the United States to Argentina, in which the presence of specific anti-*T. cruzi* antibodies has been reported [1, 29–34]. In Mexico, however, few investigations of this type have been conducted (see Table 1).

In this study, a seropositivity of 4.44% (8/180) in domestic dogs (relating to antibodies against *T. cruzi*) from an urban area in the state of Sonora was determined. In Estado de México, a direct correlation between seropositivity in humans and dogs was observed, with a rate of 7.1% in humans and 21% in dogs. In another region of the state, a high percentage of anti-*T.cruzi* IgG and IgM antibodies in dogs was found (17.5%), emphasizing the importance of dogs in the transmission route of *T. cruzi* infection [10]. This has also been observed in Palmar de Bravo, Puebla state, where a presence of 4% of anti-*T. cruzi* antibodies in humans and 10% in dogs were found [18]. A seroepidemiological study conducted in Puente Pantitlán, Morelos state reported an anti-*T. cruzi* seropositivity of 1.2% in humans and 24.2% in dogs [24]. The seropositivity rate data from Empalme was very similar to another study conducted in Chiapas (4.5%), as reported by Jiménez-Coello et al. [20]. Our results are based on the recommendations of the World Health Organization expert committee (2002), which delineate that if positive results are obtained using more than one of the three conventional tests (indirect haemagglutination, IIF, and ELISA), this can be regarded as a definitive diagnosis of *T. cruzi* infection [35].

In Sonora, 37 positive cases of CD in humans have been reported up until 2006, with a prevalence of 1.6% [7]. Although the state is identified as an endemic area, no studies have been conducted on reservoirs for the infection. For this reason, a study on the presence of *T. cruzi* in resident dogs can also offer a strong indication of the current prevalence of infection in the human population.

The seropositivity rate of Chagas infection in dogs was not related to the socioeconomic conditions of the population, nor was it influenced by the age or sex of the animal. These findings correlate with studies conducted by Jimenez-Coello et al. [19] and Martínez et al. [26], both of which were conducted in endemic areas of Mexico. However, positive samples found using the screening test (ELISA) suggest that the economic status of the geographical area may be a potential risk factor in the human and canine populations. This is supported by one dog sample (1/180, 0.55%), showing the acute state of the disease as was demonstrated by the finding of high levels of IgM, the major immunoglobuline produced during a primary immune response, which corresponds to a two-year-old male dog that belongs to an owner residing in the Pesqueira community, a northwestern locality characterized by scarce resources, poor housing conditions, and minimum municipal services, as according to INEGI data [27].

The risk of dogs from a low economic area, such as Empalme, becoming infected is possibly greater than dogs from middle or high economic zones becoming infected, as there are factors that affect the quality of life of inhabitants in these areas, such as poor housing conditions and scarce municipal services, and hence their pets are affected as well. In an endemic area of ​​Panama, significant risk factors for seropositivity for *T. cruzi* infection were found to be the type of vegetation surrounding the area and the number of domestic animals in a home, while the fitness and sex of the dogs were not found to be significant risk factors [36]. Further studies are however required to prove this hypothesis.

*Triatoma recurva* has been reported in the Mexican states of Chihuahua, Sonora, and Sinaloa, and has been found in dwellings of inhabitants of the region and in their peridomiciliary environments. The presence of *Triatoma rubida* and *Triatoma longipennis* as insects in the process of adaptation to colonize human dwellings and as important transmitter vectors of CD has been documented in the states of Jalisco, Nayarit, Colima, and Sonora [37, 38]. For these reasons, we can infer that the presence of antibodies (IgG and 1/180 IgM) against *T. cruzi* found in domestic dogs in the study area is due to an active transmission of *T. cruzi* by the vector route. There is also, however, the possibility of congenital transmission in the canine population, as has previously been reported on [28].

*Trypanosoma cruzi* epidemiology studies in domestic dogs have been performed in seven states of Mexico (see Table 1) and a direct correlation of seropositivity between humans and dogs has been found. In accordance with these data, seropositivity for *T. cruzi* in dogs in Mexico is 14.24% on average, while in endemic areas of South America, it ranges from 8% to 50% [39]. In all studies conducted in Mexico, including this one, the presence of antibodies for *T. cruzi* in dogs far exceeds that which is reported for humans, which is also consistent with reports by Gürtler et al. [39]. This may be because dogs are the preferred blood source for insects [40–42] and because dogs are commonly infected orally, a more effective infection route compared to vector transmission that usually occurs in humans [43]. Some border states of the United States have been reported to be endemic areas of the disease, and as Sonora is one of the Mexican states bordering the United States, the data provided in this study show indirect evidence of the presence of the parasite acquired by the vector route through anti-*T. cruzi* antibody detection.

The limitations of this study include the limited number of samples, the fact of not having made the search for antibodies against *T. cruzi* in the owners of the sampled dogs, as well as the study only focusing on a small region.

## Conclusions

This study shows the presence of anti-*T. cruzi* antibodies in dogs in Sonora, which suggests vector transmission. This demonstrates that in a region with heavy migration, transmission by other means such as transfusion or organ transplantation can play an important part.

This is the first study of its kind conducted in this region of Mexico. Seropositivity for *T. cruzi* in dogs owned by responsible owners (pets have the minimum conditions of care) was 4.44%, however, this could be higher for homeless dogs or dogs without owners, as these dogs would be more exposed to places where the vector could be present.

There is a need for active surveillance programs throughout the state of Sonora and vector control strategies should be implemented in endemic regions.

## Abbreviations

CD: Chagas disease
ELISA: Enzyme-linked immunosorbent assay
IgG: Immunoglobulin G
IgM: Immunoglobulin M
IIF: Indirect immunofluorescence
